# Interactions Between Phytochemicals and Minerals in *Terminalia ferdinandiana* and Implications for Mineral Bioavailability

**DOI:** 10.3389/fnut.2020.598219

**Published:** 2020-12-10

**Authors:** Saleha Akter, Michael Netzel, Ujang Tinggi, Mary Fletcher, Simone Osborne, Yasmina Sultanbawa

**Affiliations:** ^1^Queensland Alliance for Agriculture and Food Innovation, The University of Queensland, Health and Food Sciences Precinct, Coopers Plains, QLD, Australia; ^2^Queensland Health Forensic and Scientific Services, Coopers Plains, QLD, Australia; ^3^Commonwealth Scientific and Industrial Research Organisation (CSIRO) Agriculture and Food, St Lucia, QLD, Australia

**Keywords:** *Terminalia ferdinandiana*, Kakadu plum, phytate, oxalate, ascorbic acid, mineral

## Abstract

Oxalic and phytic acid are phytochemicals considered to be anti-nutritional factors as they are predominantly found as oxalates and phytates bound to minerals like calcium and potassium. Studies have associated excessive oxalate consumption with increased urinary excretion of oxalate (hyperoxaluria) and calcium oxalate kidney stone formation, and excessive phytate consumption with decreased bioaccessibility and bioavailability of certain minerals and reduced utilization of dietary protein. However, other studies suggest that dietary consumption of phytate may be beneficial and inhibit formation of calcium oxalate kidney stones. In light of these conflicting reports, dietary intake of oxalate and phytate enriched plants should be considered in relation to potential health outcomes following consumption. *Terminalia ferdinandiana* is one such plant and is investigated here with respect to oxalate, phytate, and mineral contents. Assessment of oxalate and phytate contents in *T. ferdinandiana* fruit, leaf, and seedcoat tissues through hydrolysis into acid forms revealed oxalic acid contents ranging from 327 to 1,420 mg/100 g on a dry weight (DW) basis whilst phytic acid contents ranged from 8.44 to 121.72 mg/100 g DW. Calcium content in the different tissues ranged from 131 to 1,343 mg/100 g. There was no correlation between oxalic acid and calcium, however a significant, positive correlation was observed between phytic acid and calcium (*r* = 0.9917; *p* < 0.001), indicating that tissues rich in phytic acid also contain higher levels of calcium. The high content of phytic acid in comparison to oxalic acid in *T. ferdinandiana* fruit found in this study and the dietary significance of this in terms of calcium bioavailability, needs to be investigated further.

## Introduction

*Terminalia ferdinandiana* (Kakadu plum) is a native Australian fruit consumed by Indigenous communities for centuries. In recent years, *T. ferdinandiana* has been established as a rich source of antioxidants and other biologically active compounds (1–3). Commercial demand for *T. ferdinandiana* fruit continues to increase because of its high vitamin C content (2). A variety of *T. ferdinandiana* food products are available in the market such as juices, sauces, jam and whey product isolates. Moreover, a number of *T. ferdinandiana* nutraceutical products such as energy bar (with quandong), probiotic (with manuka honey), probiotic punch (with cranberry), analgesic spray, dietary supplements (tablet and liquid format) and freeze dried powder are also available. However, a report on the high levels of oxalate (2) present in *T. ferdinandiana* fruits has raised concerns regarding the potential risk of kidney stone formation following fruit consumption. High dietary intake of oxalate is considered by some studies as the primary risk factor in the formation of calcium oxalate stones (4), however other studies have found only a modest positive correlation between stone formation and dietary intake of oxalate (5). Bioavailability of oxalate may also be influenced by the presence of *Oxalobactor formigenes* in the gastrointestinal tract (6). *O. formigenes* is one of the intestinal bacteria responsible for the degradation of oxalate in the intestine with higher levels of urinary oxalate excretion possibly due to the absence or low levels of this bacteria (6). Normal dietary intake is reported to be in the range of 50–200 mg oxalate per day but can exceed 1,000 mg per day if foods rich in oxalate are consumed.

Dietary oxalate is associated with increased urinary oxalate concentrations accounting for 50% of urinary oxalate excretion, however oxalate can also be metabolically produced by the body (7). Epidemiological evidence and short term experiments with human subjects have indicated that ascorbic acid ingestion can also serve as a risk factor for calcium oxalate stone disease and increased urinary oxalate excretion (8). Ascorbic acid (AA) and its oxidation product dehydroascorbic acid (DHAA) can form diketogulonic acid which is unstable and breaks down to oxalate (8). However, the amount of AA and DHAA that is metabolized in cells and tissues, and the amount of AA and DHAA that is converted to diketogulonic acid and ultimately oxalate, is not yet known (8). Previous studies have suggested that compromised renal function coupled with excessive AA ingestion can result in oxalate nephropathy in susceptible individuals (8).

**Figure 1:**
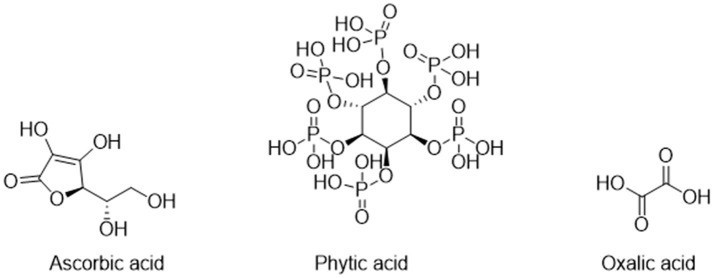
Structures of ascorbic acid (L-AA), phytic acid, and oxalic acid.

Plant phytochemicals can also affect the bioavailability of minerals. For instance, a high phytate content is associated with poor bioavailability of minerals (9). Phytate can form complexes with endogenous minerals in the intestine making them unavailable for absorption. High amounts of calcium may inhibit zinc absorption by forming insoluble calcium-zinc-phytate complexes in the intestine. However, phytic acid has also been claimed to have beneficial effects such as antioxidant properties (10) and as an inhibitor of calcium oxalate kidney stone formation (11). As a further complication, phytate can also bind proteins and reduce protein utilization (12, 13). Figure 1 illustrates the chemical structures of ascorbic acid (AA), phytic acid and oxalic acid.

Analyses of oxalate and phytate contents of *T. ferdinandiana* are necessary for informing current consumers of *T. ferdinandiana* fruit who may be at risk of kidney stone formation. Therefore, it is important to investigate and provide insights into the safety of *T. ferdinandiana* fruit products for such consumers.

This study aims to investigate certain nutritional and non-nutritional compounds in *T. ferdinandiana* fruits to provide information for determining the safe dietary intake of this fruit. For comparison, the fruits and underutilized parts, like leaves and seedcoats of *T. ferdinandiana*, are included in this study. Oxalate, phytate, and mineral contents in *T. ferdinandiana* have been evaluated along with predicted mineral bioavailability calculated as the molar ratios of phytate/oxalate/minerals (14, 15). A proximate analysis is also reported as limited studies are available on the nutrient and chemical compositions of *T. ferdinandiana* plant parts.

## Materials and Methods

### Sample Collection and Preparation

The collection of fruits has been described by Akter et al. (16). The collected fruits were sorted, washed and processed in Sunshine Tropical Fruit Products, Nambour, Queensland, Australia, to provide a seedless puree. The seeds were also collected as byproducts and were stored at −80°C until further analysis. The puree was freeze-dried (ScanVac CoolSafe Superior Touch, LabGear Australia, QLD, Australia) and ball-milled (Retsch MM400, Metrohm Australia Pty Ltd., NSW, Australia) to provide a uniform powder and stored at −20°C and used throughout this study. The frozen seeds were thawed and washed with distilled water to remove the pulp and peel residue. The seeds were then oven-dried for 48 h at 40°C. After drying, the seeds were individually cracked using an Engineers vice size 125 (DAWN tools and Vices Pty Ltd., Heidelberg West, Victoria, Australia) to release the kernels from the seedcoats. The kernels were processed and analyzed separately in a previous study (17). The separated seedcoats were hammer milled and used for this study. Mature leaves were collected from ten trees (ten leaves from each tree) from the same region during the same fruit harvest and were freeze-dried and milled together to prepare a composite leaves samples. The milled freeze-dried powders of leaves were used throughout this study.

### Proximate Composition Analysis

A complete proximate analysis was performed in a National Association of Testing Authorities (NATA) accredited laboratory that complies with ISO/IEC17025:2005, Symbio Alliance (Eight Mile Plains, Queensland, Australia) using AOAC standard methods (18). Moisture was measured (AOAC 925.10) by air oven with a measurement of uncertainty (MU) of ±15%, ash (AOAC 923.03), crude protein (AOAC 990.03) by Dumas combustion with a MU of ±10%, dietary fiber by (AOAC 985.29) with a MU of ±15%, crude fiber by (AOAC 962.09), crude fat (AOAC 991.36) with a MU of ±15%, and saturated, mono-unsaturated, poly-unsaturated and trans fat by in house method CFH068.2. Carbohydrate and energy by calculation using information from the Food Standards Australia New Zealand (FSANZ) Code.

### Mineral and Trace Element Composition Analysis

Mineral and trace element levels of *T. ferdinandiana* fruit, leaf and seedcoat were determined based on the methods described previously (17) and expressed on a dry weight basis. Briefly, samples (ca. 0.3 g) were subjected to overnight slow digestion at room temperature and followed by microwave digestion (MarsXpress, CEM, Matthews, NC, USA) at a gradual increase in temperature. The levels of minerals were analyzed by inductively coupled plasma optical emission spectrometry (ICP-OES, 700 Series, Agilent, VIC, Australia) and the trace elements were analyzed using ICP-MS for greater sensitivity on a 7700 instrument (Agilent, Tokyo, Japan). Operating conditions and parameters of ICP-OES were recorded as follows: operating power 1.20 KW, plasma gas flow rate 15.0 L/min, auxiliary gas flow rate 1.50 L/min, nebuliser gas flow rate 0.90 L/min, sample uptake 50 s, pump rate 25 rpm and rinse time 30 s. The elements were recorded at the following wavelengths (nm): Ca 370.602 (nm), K 766.491 (nm), Mg 383.829 (nm), Na 568.821 (nm) and P 185.878 (nm). Operating conditions of the ICP-MS were recorded as follows: radio frequency power 1,350 W, carrier gas 0.8 L/min (argon) and gas flow rate 4.5 mL/min (helium reaction cell). Standard reference materials were used for the quality control and were treated similarly to the samples (19).

### Phytate Content Determination

*T. ferdinandiana* plant parts were analyzed for phytic acid content by a previously described method with slight modification (20). Samples of fruits, leaves and seedcoat powder (0.5–1.0 g) were weighed and mixed with 2.4% HCl (20 mL). The samples were vortexed and placed in a rotary mixer for 16 h at room temperature at low speed. The sample mixtures were centrifuged at 5,000 rpm for 10 min at 10°C (Eppendorf centrifuge 5810R). The supernatants were transferred to 50 mL centrifuge tubes containing 1 g of NaCl and rotary mixed for 20 min at room temperature. The tubes were allowed to settle at 4°C for 1 h and then centrifuged at 5,000 rpm for 20 min at 10°C. Aliquots of these supernatants (100 μL) were diluted with 1,900 μL of RO water in 75 mm culture tubes. Phytic acid sodium salt hydrate purchased from Sigma-Aldrich (St. Louis, MI, USA) (0–50 μg/mL) was used as a standard dissolved in RO water. An aliquot (900 μL) of both standards and diluted supernatants of the samples were then mixed with 300 μL of Wade's reagent (0.03% FeCl_3_.6H_2_O + 0.3% Sulfosalicylic acid) in 2 mL microfuge tubes and vortexed. The absorbance was recorded at 500 nm (21) using a DU 530 UV-Vis spectrophotometer (Beckman Coulter Inc. Brea, CA, USA). An external calibration curve was prepared from standard phytic acid sodium salt hydrate to calculate the phytic acid concentrations in the samples. The results were multiplied by 0.282 (molar ratio of phytate—phosphorous in a molecule of phytate) in order to express the phytate content in the sample as mg/100 g of phytate on a dry matter basis (22).

### Oxalate Content Determination

Extraction and quantification of water-soluble and total oxalate were performed according to a previously described method (2). Oxalate compounds were converted to oxalic acid using hydrochloric acid and analyzed on a Phenomenex HPLC system (Phenomenex, Lane Cove West, NSW, Australia) equipped with a Gilson UV/VIS 151 multiwavelength detector (Gilson, Middleton, WI, USA). A Phenomenex reversed phase column (Synergi 4 μm Hydro-RP 80A, 250 × 4.60 mm) (Phenomenex, Lane Cove West, NSW, Australia) was used with an isocratic mobile phase of 20 mM phosphate buffer (pH 2.4) and a flow rate of 0.5 ml/min. The injection volume was 20 μL and detection was carried out at 210 nm. Sodium oxalate was obtained from Sigma-Aldrich (St. Louis, MI, USA). Sodium oxalate at the concentrations of 1, 2, 3, 4, and 8% in 20 mM phosphate buffer (pH 2.4) was used to prepare an external calibration curve to quantify the levels of total and water-soluble oxalate in *T. ferdinandiana* tissue samples.

### Ascorbic Acid Determination

Vitamin C extraction was performed by following previously described method (23, 24) with modification. Briefly, 0.5 g powders of *T. ferdinandiana* fruit, leaves and seedcoat was added to 5 ml of freshly prepared extraction solution composed of 3% metaphosphoric acid (MPA) (Merck, Darmstadt, Germany), 8% acetic acid (Merck, Darmstadt, Germany) and 1 mM ethylenediaminetetraacetic acid tetrasodium salt (EDTA) (Merck, Darmstadt, Germany). The homogenate was thoroughly vortexed and then centrifuged at 10,000 rpm (refrigerated at 2–4°C) for 10 min. The supernatant was collected, the extraction was repeated twice and supernatants were pooled together. Samples for ascorbic acid (AA) analysis were prepared according to a previously described method (23). Briefly, 500 μL of extracted solution was added to 500 μL of freshly prepared 0.5 M Trizma buffer (Merck, Darmstadt, Germany). An aliquot (100 μL) of formic acid (Sigma-Aldrich, Castle Hill, NSW, Australia) was added to the mixture. In an UPLC vial, 900 μL of milli-Q water and 100 μL of the sample mixture was added. Samples for dehydroascorbic acid (DHAA) analysis were prepared by adding 500 μL of extracted solution to 500 μL of 40 mM DTT (DL-Dithiothreitol) (Sigma-Aldrich, Castle Hill, NSW, Australia) in Trizma buffer. The mixture was vortexed and left in the dark at room temperature for 30 min. In an UPLC vial, 900 μL of milli-Q water and 100 μL of the sample mixture was added. The DHAA content of the samples were calculated by subtracting the initial AA content from the total vitamin C content, after reduction with DTT. Oxidation of AA from standard solutions and samples were prevented by performing the experiment under reduced light, using amber flasks and vials, minimal oxygen exposure and in low temperature. DHAA was quantified by the difference between the total AA content (after DHAA reduction to AA) and the AA contents before the DHAA conversion (23). A standard stock solution of AA (Sigma-Aldrich, Castle Hill, NSW, Australia) (ca. 2 mg/10 mL) in milli-Q water was prepared fresh on each day of analysis and was stored in amber flasks at 4°C prior to chromatographic analysis. Solutions of variable concentrations were prepared by diluting the standard stock solution with milli-Q water. An Acquity UPLC system (Waters Corp., Milford, MA, USA), equipped with a Waters Acquity UPLC photodiode array (PDA) detection system was used to carry out the analysis. Empower™ software (Waters Corp., Milford, MA, USA) was used to process and quantify peaks after recording the signals. An Acquity HSS T3 analytical column (100 × 2.1 mm, 1.8 μm particle size) (Waters Corp., Milford, MA, USA), using an isocratic mobile phase 0.1% aqueous (v/v) formic acid at a flow rate of 250 μL/min was used with an injection volume of 2 μL. The absorbance was measured at room temperature at 245 nm using PDA.

### Estimation of the Potential Bioavailability of Minerals

The molar ratios have been calculated by using the following formula (25):

$$\begin{array}{l} Phytate\;to\;mineral\;molar\;ration=\frac{Phytate\;\left(mg\right)/atomic\;weight\;of\;phytate}{Mineral\;\left(mg\right)/atomic\;weight\;of\;minerals}. \end{array}$$

### Statistical Analysis

GraphPad Prism version 8 (GraphPad Software, San Diego, California, USA) was used to analyse the data. One-way ANOVA was performed with Tukey's multiple comparison test to evaluate the significance of differences between groups and to compare between groups. *P-*values ≤ 0.05 was considered as statistically significant. Regression analyses were performed to determine the correlation (pearson *R*^2^) between oxalate vs. phytate, oxalate vs. calcium and phytate vs. oxalate contents of the plant parts. Data are reported as mean ± SD of three measurements unless otherwise specified.

## Results and Discussion

### Proximate Composition

The results of the proximate analysis are summarized in Table 1. Protein, fat, ash and energy content were highest in leaves followed by fruits and seedcoats. Seedcoats contains the highest amount of dietary (90.2%) fiber. Moisture content of fruits is higher than leaves and seedcoats. Study on the proximate composition of the kernels of *T. ferdinandiana* showed that kernels contain 32% protein, 35% fat and is a good source of minerals and nutrients (17). Studies on the proximate composition of *T. ferdinandiana* fruits, leaves and seedcoats are scarce and hence no values are available for comparison. The high dietary fiber content in seedcoats could potentially be a functional food ingredient to improve dietary fiber intake and help reduce hypercholesterolemia, gallstone, constipation, diabetes, coronary heart disease, and various digestive disorders (26).

**Table 1:** Proximate composition of *T. ferdinandiana*.

		***T. ferdinandiana*** **plant parts**		
		**Fruits**	**Leaves**	**Seedcoats**
Protein	%	4.7	7.1	2.3
Fat	%	0.9	6.5	0.6
Saturated Fat	%	0.3	2.5	0.1
Mono-unsaturated Fat	%	<0.1	0.4	0.2
Poly-unsaturated Fat	%	0.6	3.7	0.4
Trans Fat	%	<0.01	<0.01	<0.01
Ash	%	5.5	5.9	0.6
Moisture	%	6.0	3.0	2.2
Dietary Fiber (Total)	%	45.9	44.0	90.2
Energy	KJ/100 g	1,110	1,283	853
Total Sugar	g/100 g	2.3	2.5	<0.10
Available Carbohydrate	%	37.0	33.4	4.0

*Results are expressed as mean of triplicate experiments on a dry weight basis. Means are not significantly different (p = 0.96)*.

### Mineral Composition

The levels of major minerals and trace elements in *T. ferdinandiana* are presented in Table 2. Calcium content was found to be higher in leaves [1,343 mg/100 g on a dry weight (DW) basis] compared to fruits (295 mg/100 g DW) and seedcoats (131 mg/100 g DW). Seedcoats were found to contain low levels of all major minerals compared to fruits and leaves consistent with the ash content of *T. ferdinandiana* plant parts included in Table 1. The findings here are similar to previous studies that report the calcium content of freeze dried *T. ferdinandiana* fruits to be 282 mg/100 g DW (33) with an average content of individual whole fruit to be 243 mg/100 g DW (2). Potassium content in *T. ferdinandiana* fruits was found to be 2,718 mg/100 g DW in this study and is more than 30% higher than previously reported levels (1,906 mg/100 g DW) (33). Phosphorus content of the fruits was found to be 73 mg/100 g DW and is also higher than a previous study (52.5 mg/100 g DW) (33).

**Table 2:** Mineral composition of *T. ferdinandiana*.

	**Fruits**	**Leaves**	**Seedcoats**	**Dietary reference intakes**	
	**mg/100 g DW**	**mg/100 g DW**	**mg/100 g DW**		**Units**
**Major elements**					
Ca	295 ± 0.6	1,343 ± 14	131 ± 19	1,200 AI (27)	mg/person/day
K	2,718 ± 99	1,179 ± 22	265 ± 0.4	4.7 AI (27)	g/person/day
Mg	204 ± 0.7	403 ± 2	24 ± 4	350 EAR (27)	mg/person/day
Na	212 ± 43	202 ± 80	96 ± 11	1.3 AI (27)	g/person/day
P	73 ± 2	64 ± 2	20 ± 4	700 RDA (27)	mg/person/day
**Trace elements**					
Fe	1.7 ± 0.0	3.4 ± 0.1	3.9 ± 0.7	8 RDA (27)	mg/person/day
Zn	2.2 ± 1.6	2.0 ± 0.1	0.3 ± 0.0	11 RDA (27)	mg/person/day
Co	0.01 ± 0.0	0.09 ± 0.0	<0.01	0.12AI (27)	μg/person/day
Ni	0.5 ± 0.0	0.1 ± 0.0	0.04 ± 0.0	1.0 UL (27)	mg/person/day
Cu	1.4 ± 0.0	0.6 ± 0.0	1.0 ± 0.1	700 RDA (27)	μg/person/day
Cr	0.07 ± 0.0	0.02 ± 0.0	0.02 ± 0.0	35 AI (27)	μg/person/day
Mn	5.0 ± 0.0	25.5 ± 0.7	1.3 ± 0.0	2.3 AI (27)	mg/person/day
Sr	4.0 ± 0.0	13.0 ± 0.0	1.4 ± 1.4	1–5 RDA (27)	mg/person/day
Mo	0.04 ± 0.0	0.01 ± 0.0	<0.1	34 EAR (27)	μg/person/day
Se	<0.01	<0.01	<0.1	45 EAR (27)	μg/person/day
B	2.0 ± 0.1	3.5 ± 0.0	0.4 ± 0.1	20 UL (27)	mg/person/day
Ba	2.3 ± 0.0	2.8 ± 0.0	0.7 ± 0.0	0.02 UL (28)	mg/kg BW
**Heavy metals**					
As	<0.01	<0.01	<0.1	12.5–25 UL (29, 30)	μg/kg BW/week
Cd	<0.01	<0.01	<0.1	2.5 UL (30, 31)	μg/kg BW/Week
Hg	<0.01	<0.01	<0.1	5 UL (30, 32)	μg/kg BW/week
Pb	<0.01	<0.01	0.04 ± 0.0	25 UL (30, 32)	μg/kg BW/week

*Data are presented as mean ± SD of duplicate determinations. Means are not significantly different (p = 0.46). RDA, recommended dietary allowance; AI, adequate Intake; UL, tolerable upper intake level; EAR, Estimated average requirement; BW, body weight*.

The contribution of *T. ferdinandiana* to daily intakes of minerals and trace elements can be assessed based on the guideline on daily requirements of essential elements as recommended for adults. The average daily adult requirements for trace elements (in men and women aged 19–70 years) are as follows: Fe 8–18 mg/day; Zn 8–14 mg/day; Cu 1.2–1.7 mg/day; and Mn 5–5.5 mg/day (34). *T. ferdinandiana* fruits contain 1.7 mg/100 g DW of Fe that is much lower than some grains such as sorghum 3.7 mg/100 g DW, soybean 7.3 mg/100 g DW, and mung bean 7.2 mg/100 g DW (35). Zinc content of *T. ferdinandiana* fruit is 2.2 mg/100 g DW and is higher than a value (0.6 mg/100 g DW) previously reported (33). *T. ferdinandiana* fruits have lower levels of Zn compared with other common legumes and grains such as soybean (3.6 mg/100 g DW), mung bean (2.8 mg/100 g DW), rice (2.9 mg/100 g DW), and millet (3.7 mg/100 g DW) (35). Manganese levels in *T. ferdinandiana* leaves are much higher (25.5 mg/100 g DW) compared to fruits (5.1 mg/100 g DW) and seedcoats (1.3 mg/100 g DW).

The heavy metal content of *T. ferdinandiana* (Table 2) are <0.1 mg/100 g DW for all metals tested except for Pb content in the seedcoats that was found to be 0.04 mg/100 g DW. From our present study, it can be inferred that *T. ferdinandiana* fruit presents no known risk for heavy metal toxicity if 1 g freeze dried powder is consumed per day. It is important to note that differences in geographical variables such as soil fertility, mineral uptake efficiency of plants, and growth conditions may cause variation in the heavy metal concentrations in *T. ferdinandiana* (33, 36).

### Phytate Content

**Figure 2:**
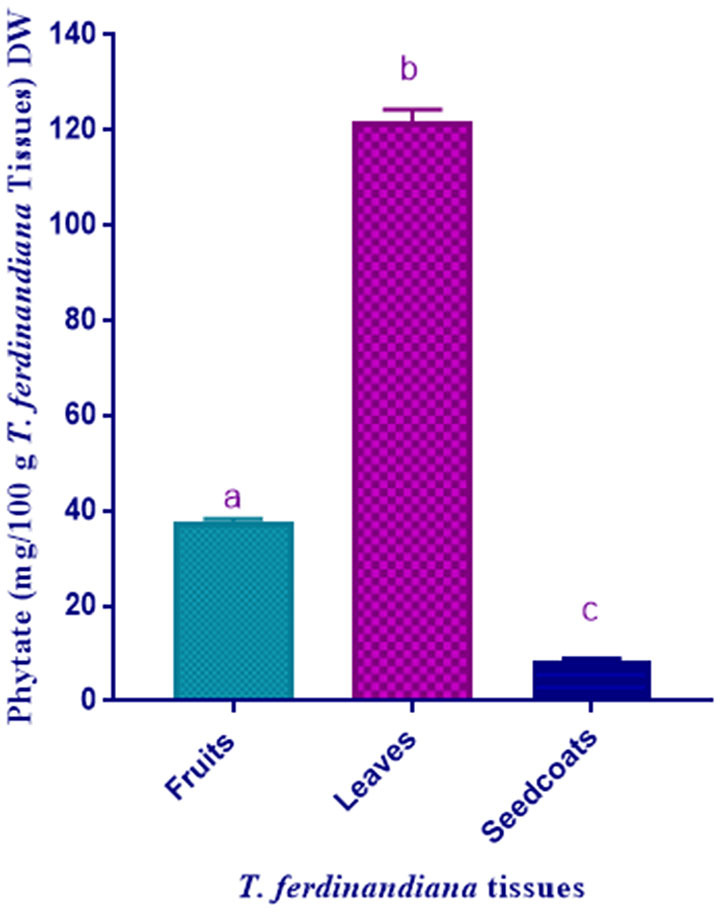
Phytate content of *T. ferdinandiana*. Results are shown as the mean of triplicate experiments ± SD. Different lower case letters indicate significant differences (*P* ≤ 0.05).

Phytate contents, measured as phytic acid following hydrolysis of phytate, in *T. ferdinandiana* plant parts are presented in Figure 2. Leaves were found to contain the highest amount of phytate (121.72 mg/100 g DW) compared to fruits (37.6 mg/100 g DW) and seedcoats (8.44 mg/100 g DW). Typical values for phytic acid content in cereal foods are in the range of 0.5–1% by weight (37). The values obtained in the present study are lower than plants and grains previously reported to contain high phytate levels. For example, phytate levels range from 1,000 to 2,200 mg/100 g DW in soybeans and from 590 to 1,100 mg/100 g DW in mung beans (35). The phytate levels of some seeds and grains were also reported to be high such as rice 1,084, cowpea 559, maize 908, sorghum 925, and soybean 878 mg/100 g DW (35). Globally, daily intake of phytic acid varies largely according to diet from ~0.2–4.6 g. Vegetarian diets generally contains higher amounts of phytic acid compared to mixed diets (37). Several methods have been developed so far to reduce the phytic acid content in food owing to the anti-nutritional effect and to improve the nutritional value of the food. Several pre-treatment methods such as fermentation, soaking, germination and enzymatic treatment of grains with phytase enzyme can be applied besides genetic improvement (9). On the other hand, researchers have also suggested that soy foods and soybeans can be advantageous for kidney patients due to the inhibition of calcium kidney stone formation with high concentrations of phytate (38). The Phytate content determination by spectroscopic method can sometimes cause overestimation if the plant material also contains oxalate. Krome et al. have shown that determining phytate contents by using photometric method require correction due to the fact that the Wade-reagent interacts with oxalate and phytate present in plant material (39). Extensive literature search in support of the existing issue of overestimation has showed us that overestimation of phytate has been documented before (36, 37).

In the spectroscopic method, the pink color of the Wade reagent is due to the reaction between the ferric ion and the sulfosalicylic acid. In the presence of phytic acid, the ferric ion binds to the phosphate ester and is unavailable to react with the sulfosalicylic acid, resulting in a decrease of the pink colors intensity. Determination of the phytic acid level by the spectrophotometric method can show higher phytic acid levels (27% in some cases) than the chromatographic methods (40). The researchers have shown that the cause of phytate overestimation with methods involving the Wade reagent lies in other anions present in the samples predominantly oxalate. They have suggested that the magnitude of the error of a phytate determination involving the Wade-reagent therefore depends on the concentration of these compounds in the respective plant material. However, they have suggested that this method should only be applied if the concentration and their degree of influence on the analysis is known (39). To overcome the ongoing issue of overestimation of phytate content by using colorimetric assay, an appropriate anion-exchange chromatography combined with colorimetric method was suggested by researchers (40, 41). Considering the limitation of the colorimetric assay, future work has been designed to determine the phytate levels in *T. ferdinandiana* plant parts by employing an anion-exchange chromatography combined with colorimetric assay. A comparative study will also be performed on the levels of phytic acid in the *T. ferdinandiana* plant parts obtained from colorimetric assay and anion-exchange chromatography combined with colorimetric assay.

### Oxalate Content

Oxalate contents (measured as oxalic acid following hydrolysis of total and soluble oxalate) in *T. ferdinandiana* plant parts are presented in Table 3. Among the three parts analyzed, fruits were found to contain the highest levels of oxalate followed by leaves. Total oxalate content in seedcoats was significantly lower than leaves and fruits. The content of total and soluble oxalate varied widely between the three different parts with variations more evident when soluble oxalate was expressed as a percentage of total oxalate (Table 3). The greatest proportion of soluble oxalate was in seedcoats (96%) followed by fruits (79%) and leaves (32%). Previous reports of oxalate levels in *T. ferdinandiana* fruits and leaves were 2,717 mg/100 g DW and 1,636 mg/100 g DW and are much higher than the values obtained in our present study (2). Variables including, but not limited to, geographical location, soil fertility, growth conditions, methodological aspects and extraction techniques may cause variation in the oxalate concentrations compared with previous reports.

**Table 3:** Oxalate levels in *T. ferdinandiana*.

***T. ferdinandiana* plant parts**	**Fruits**	**Leaves**	**Seedcoats**
Total oxalate (mg/100 g DW)	1,420 ± 105^a^	1,133 ± 131^b^	327 ± 15^c^
Water-soluble oxalate (mg/100 g DW)	1,120 ± 141^a^	360 ± 20^b^	313 ± 35^b^
Soluble as % of Total	79	32	96

*Results are presented as mean ± SD of triplicate determinations. Numbers with different superscripts^a, b, c^ in the same row are significantly different (p ≤ 0.05) as determined by one-way ANOVA and Tukey's multiple comparison test*.

Foods are considered high in oxalate when levels are >50 mg/100 g DW (42). The American Dietetic Association recommends that patients with kidney stones should restrict dietary intake of oxalate to <40–50 mg per day (42). High oxalate containing foods include spinach (400–900 mg/100 g FW), legumes, rhubarb raw (275–1,336 mg/100 g FW), cereals, beets (roots and leaves), chocolate, some tree nuts, star fruit raw (80–730 mg/100 g FW), black teas (not green or herbal), and bran concentrates (42–44). The results of the present study indicate that the serving suggestion of 1 g of freeze-dried powder of *T. ferdinandiana* per day may provide 14.2 mg total oxalate. This value is lower than the recommended dietary intake of oxalate for patients with kidney stones (42). The recommended serving size for green leafy vegetables, such as spinach is 1 metric cup (ca. 30 g) (45) that contains at least 270 mg total oxalate calculated from published data (42). However, caution should be taken by kidney stone patients, and patients with increased risk of kidney disease, when consuming any foods high in oxalate.

**Figure 3:**
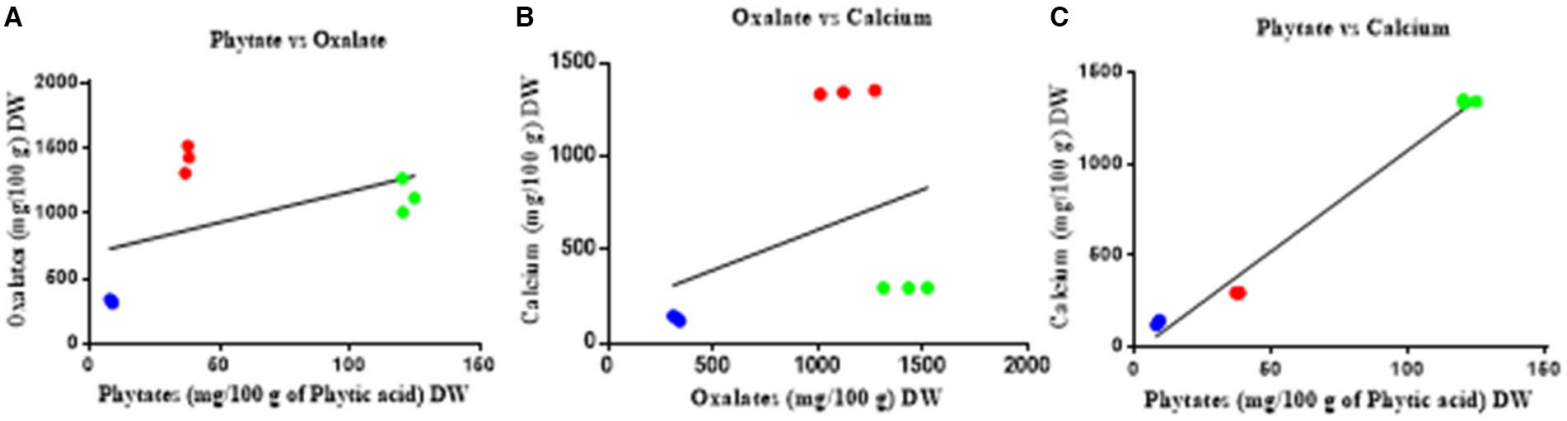
Correlation between oxalate, phytate, and calcium of *T. ferdinandiana*. **(A)** Phytate vs. oxalate, **(B)** oxalate vs. calcium, and **(C)** phytate vs. calcium in fruits 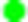, leaves 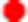, seedcoats 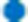. No significant associations were found except with phytate vs. calcium (pearson *r* = 0.9917; *p* < 0.001).

There is no significant correlation between calcium and oxalate (Figure 3) or between oxalate and phytate, however there is a significant correlation between phytate and calcium (*r* = 0.9917; *p* < 0.001) across all parts of the plant.

### Vitamin C Content

The contents of AA, DHAA, and total vitamin C in freeze dried *T. ferdinandiana* fruits was 16.4 ± 0.7, 4.7 ± 2.8, and 21.1 ± 2.2 g/100 g DW, respectively. The total vitamin C content (AA and DHAA together) in food products is used as an index of quality because it is very sensitive to degradation during processing and storage (23). Therefore, simultaneous analysis of AA and DHAA is necessary in food analysis to determine the total vitamin C content, as DHAA exhibits equivalent biological activity to AA (23). In a previous report, total vitamin C levels in whole fruit was 18.16 g/100 g DW (2) and in composite fruit extracts it was 14.04 g/100 g DW (46). This variation could be due to wild harvesting of fruits occurring in different geographical locations, climatic and soil conditions and seasonality. However, it is also important to determine the accurate content of vitamin C in foods to better understand the relationship of dietary intake and human health (47). National Health and Medical Research Council Australia has recommended the Recommended Dietary Intake (RDI) of vitamin C for healthy adult males and females as 45 mg/day and a upper level of intake (UL) is not possible to establish with certainty for supplementary vitamin C however a 1,000 mg/day can be a prudent limit (48). Based on the result of this study, having 1 g of freeze dried *T. ferdinandiana* powder everyday would fulfill the RDI (100%) for Vitamin C and will be within the 1,000 mg/day prudent limit.

### Molar Ratios to Predict the Potential Bioavailability of Minerals

From a nutritional point of view, bioavailability can be defined as the fraction of an ingested component available for utilization in normal physiological functions (49). Calcium bioavailability can be influenced by the presence of phytic acid through the formation of calcium-phytate complexes that limit the number of free calcium able to bind with oxalate (potentially forming calcium oxalate kidney stones). Many techniques have been employed to determine the bioavailability of minerals from foods to assess mineral utilization in the human body (50). Measuring the molar ratio of phytate/minerals and oxalate/minerals can predict the bioavailability of minerals (25) and the result obtained from this method is only relative.

Critical molar ratio values are used as guideline to assess mineral bioavailability. The critical values for phytate:calcium is >0.24 (26), phytate:iron >1 (15), phytate:zinc >15 (51). If molar ratios are close to these values, a normal homeostasis can be maintained. However, if molar ratios are higher than these values, the presence of phytate can alter the bioavailability of minerals. Molar ratios of phytate to minerals >0.24 and oxalate to minerals >2 have been reported as unsafe (15). Oxalate to mineral ratio >2 indicates that excess oxalate is bioavailable and could limit mineral absorption (15).

The molar ratio of phytate: calcium present in the fruits and leaves is 0.01 and in seedcoats < 0.01 (Table 4). The results suggest that phytate level in *T. ferdinandiana* are within the safe level and it can be suggested that the presence of phytate would not decrease the availability of calcium in *T. ferdinandiana*. According to some previous reports, the molar ratio of phytate: calcium for some commonly consumed food items such as kidney beans is 0.53, white bean 0.76, wheat bran 8.89, honey coated cereal 0.01, cooked white rice 1.65 and wheat flour 3.07 that are comparable to the phytate: calcium ratio of *T. Ferdianadiana* (15, 51). The molar ratio of phytate: magnesium in the fruit, leaves and seedcoats is 0.01 which is also within the safe level (Table 4). The ratios of phytate: iron in the *T. ferdinandiana* fruit is 1.87, leaves 3.03 and seedcoats 0.18, respectively. Phytate: zinc molar ratios >15 is indicative of poor zinc bioavailability (26). The phytate: zinc molar ratios of *T. ferdinandiana* (1.69–6.03) are within the safe level. Based on the results, it can be stated that phytate would not impact the bioavailability of zinc in the *T. ferdinandiana*. Moreover, the phytate: zinc molar ratios of soybean is 23.5, cowpea 15.8, sorghum 62.8, and maize 40.6 (35) which are much higher than the ratio of *T. ferdinandiana*.

**Table 4:** Molar ratios of phytate, mineral, and oxalate in *T. ferdinandiana*.

	**Fruits**	**Leaves**	**Seedcoats**
Phytate: Iron	1.87	3.03	0.18
Phytate: Zinc	1.69	6.03	2.79
Phytate: Calcium	0.01	0.01	<0.01
Phytate: Magnesium	0.01	0.01	0.01
Phytate: Potassium	<0.01	0.11	0.02
Oxalate: Calcium	1.51	0.26	0.78
Oxalate: Magnesium	1.32	0.53	2.59
Oxalate: Potassium	0.16	5.40	4.99
Phytate: Oxalate	0.01	0.02	0.01

The molar ratio of oxalate: calcium present in the fruits is 1.51, seedcoats 0.26 and leaves 0.78 in Table 4. The results suggest that oxalate level in *T. ferdinandiana* are within the safe level as oxalate to mineral molar ratio of >2.0 indicates unsafe (15). Similarly, the molar ratio of oxalate: magnesium is within the safe level for fruit (1.32) and leaves (0.53) and slightly high in seedcoats (2.59) (Table 4). The molar ratio of oxalate:calcium for some commonly consumed food items such as oat bran is 3.14, barley bran 3.14, red kidney bean 0.95 and white bean 0.28, respectively (15).

## Conclusion

Recent research on the *T. ferdinandiana* have revealed information about the biological activities of phytochemicals present in *T. ferdinandiana* as well as the functions of *T. ferdinandiana* in food formulations and applications. In the present study, the proximate and mineral composition, vitamin C, phytate and oxalate contents of *T. ferdinandiana* have been investigated. This is the first study to report the phytate levels in *T. ferdinandiana* as well as in providing a detailed report on the nutritional and anti-nutritional compositions of *T. ferdinandiana*. The findings of this study will potentially increase consumer knowledge on the safe use of *T. ferdinandiana*. Phytate is considered anti-nutritional due to the chelating action of essential minerals. Results of the present study suggests that the phytate present in *T. ferdinandiana* would not have an impact on the availability of essential minerals, especially calcium. The oxalate calcium molar ratio is also within a safe level. However, predictive bioavailability determinations need to be confirmed by various *in vitro* and *in vivo* studies before making any claim on the safety of these anti-nutritional compounds. Future investigations into the *in vitro* bioaccessibility and bioavailability of oxalate, phytate, and calcium in *T. ferdinandiana* should be performed to better understand the implications of the anti-nutritional factors for health.

## Data Availability Statement

The original contributions presented in the study are included in the article/supplementary materials, further inquiries can be directed to the corresponding author/s.

## Author Contributions

SA, MN, MF, and YS conceived and designed the study. SA performed the experiments, analyzed data, and wrote the manuscript. UT helped in analyzing the mineral contents. MN, UT, SO, MF, and YS critically revised and edited the manuscript. All authors contributed to the article and approved the submitted version.

## Conflict of Interest

The authors declare that the research was conducted in the absence of any commercial or financial relationships that could be construed as a potential conflict of interest.
